# Natural Herbal Medicine as a Treatment Strategy for Myocardial Infarction through the Regulation of Angiogenesis

**DOI:** 10.1155/2022/8831750

**Published:** 2022-05-12

**Authors:** Mu-xin Zhang, Yu Song, Wan-li Xu, Ling-xiao Zhang, Chao Li, Yun-lun Li

**Affiliations:** ^1^First Clinical Medical College, Shandong University of Traditional Chinese Medicine, Jinan 250355, China; ^2^Innovation Research Institute of Traditional Chinese Medicine, Shandong University of Traditional Chinese Medicine, Jinan 250355, China; ^3^College of Traditional Chinese Medicine, Shandong University of Traditional Chinese Medicine, Jinan 250355, China; ^4^Department of Cardiology, The Affiliated Hospital of Shandong University of Traditional Chinese Medicine, Jinan 250014, China

## Abstract

**Methods:**

We conducted a literature search on the bioactive components of medicinal plants and their effects on angiogenesis after MI. We searched for articles in Web of Science, MEDLINE, PubMed, Scopus, Google Scholar, and China National Knowledge Infrastructure databases before April 2021.

**Results:**

In this article, we summarized the mechanisms by which copper ions, microRNA, Akt1, inflammation, oxidative stress, mitochondria, and pericytes are involved in angiogenesis after myocardial infarction. In addition, we reviewed the angiogenic effects of natural herbal medicines such as *Salvia miltiorrhiza* Bunge Bunge, *Carthamus tinctorius* L., *Pueraria lobata*, *Astragalus*, *Panax ginseng* C.A. Mey., *Panax notoginseng* (Burkill) F.H. Chen, *Cinnamomum cassia* (L.) J. Presl, *Rehmannia glutinosa* (Gaertn.) DC., *Leonurus japonicus* Houtt, *Scutellaria baicalensis* Georgi., and *Geum macrophyllum* Willd.

**Conclusions:**

Some herbs have the effect of promoting angiogenesis. In the future, natural proangiogenic drugs may become candidates for the treatment of cardiovascular diseases.

## 1. Introduction

Myocardial infarction (MI) occurs with insufficient blood supply to the coronary arteries and is usually caused by coronary artery stenosis or occlusion [1]. In 2017, the global incidence of MI due to ischemic heart disease was 10.636.5 million [2]. According to World Health Organization, MI has become the number one cause of death in the world and places a great burden on human lives. Therefore, the prevention and treatment of MI have become a major focus of attention.

Of the many ways to treat MI, one of the most basic is the application of drugs, such as aspirin, nitroglycerin, angiotensin-converting enzyme inhibitors (ACEI)/angiotensin receptor blocker (ARB), beta-blockers, diuretics, and statins [3]. At present, the most effective and most clinically applied strategies for MI are percutaneous coronary intervention (PCI) and coronary artery bypass graft (CABG), which aim to increase the blood supply of the ischemic myocardium [4]. However, these methods have higher requirements on the patient's physical condition; e.g., patients who are allergic to iodinated contrast agents or with diffuse coronary artery stenosis cannot undergo the above-mentioned surgeries. In addition, even if the operation is successful, the MI of patients undergoing CABG can only be reduced by an average of 30% [5], and the incidence of patients undergoing PCI with postoperative residual disease and ischemia-reperfusion without reflow was 46% [6]. Therefore, the above treatments have many shortcomings, so a new treatment direction is urgently needed to deal with MI.

“Therapeutic angiogenesis” is considered to be the most valuable and promising complementary treatment for MI [7]. The new blood vessels can improve local microcirculation, restore the blood supply in ischemic areas, and fundamentally relieve MI. With the induction of ischemia, existing capillaries at the site of MI are exposed to angiogenesis-stimulating factors, and endothelial cells are detached from the blood vessel wall, leading to increased vascular permeability. Then, endothelial cells migrate to the temporary matrix formed by the degradation of basement membranes and extracellular matrix. Finally, a vascular endothelial cell membrane is formed, which wraps pericytes and integrates them into the blood circulation to complete angiogenesis [8]. However, due to the release of proinflammatory mediators, the lack of proangiogenic factors, and other reasons, physiological angiogenesis is slow [9], and neither the number nor size of the new blood vessels provides enough blood supply for the ischemic heart muscle. Therefore, the role of therapeutic angiogenesis is self-evident. Unfortunately, there is a lack of drugs that can increase angiogenesis.

Natural herbal medicines, which have the advantages of being multitarget and multicomponent, few side effects, and low cost, are attracting increasing attention. It has been reported that many biologically active compounds from natural sources were powerful inducers of angiogenesis [10], including *Salvia miltiorrhiza* Bunge, *Carthamus tinctorius* L, *Pueraria lobata*, *Astragalus*, *Panax ginseng* C.A. Mey., *Panax notogins*eng (Burkill) F.H. Chen, *Cinnamomum cassia* (L.) J. Presl, *Rehmannia glutinosa* (Gaertn.) DC, *Leonurus japonicus* Houtt, *Scutellaria baicalensis* Georgi., and *Geum macrophyllum* Willd. In this review, we will focus on the pathological mechanism underlying disordered angiogenesis after myocardial infarction and review natural herbal drugs that promote angiogenesis.

## 2. Method and Strategy

For this review, we conducted a literature search on the bioactive components of medicinal plants and their effects on angiogenesis after MI. We searched for articles in Web of Science, MEDLINE, PubMed, Scopus, Google Scholar, and China National Knowledge Infrastructure databases before April 2021 using to the following keywords and phrases: “natural drugs and angiogenesis,” “natural active ingredients and angiogenesis,” “phytochemistry/medicinal plant extracts and angiogenesis,” “medicinal plants and myocardial infarction,” and “natural active ingredients and myocardial infarction.” From the search results, we selected original papers that discussed the effects of natural herbal medicines or their active ingredients on therapeutic angiogenesis after MI.

## 3. Angiogenesis

In the 19th century, the concept of “angiogenesis” was first proposed by John Hunter, a Scottish doctor. At that time, people realized that the formation of new blood vessels was a key step in tissue expansion and repair. Angiogenesis plays an important role in growth and development, tissue regeneration, vascular repair, and many pathological conditions. Embryonic development and wound healing after myocardial infarction and fracture are inseparable from angiogenesis [11]. However, blocking angiogenesis to slow down the growth and progression of tumors is a standard treatment for various cancers [12]. In addition, diseases such as arthritis, endometriosis, and macular degeneration also require antiangiogenesis [13].

During angiogenesis, due to relative ischemia, hypoxia, inflammation, oxidative stress, and other factors, vascular endothelial growth factor (VEGF), endothelial cells, and pericytes are induced to generate new blood vessels [14]. In the process of myocardial ischemia, the gene encoding hypoxia-inducible factor-1 (HIF-1) is first upregulated, enhancing the expression of VEGF-A. VEGF-A is the main hypoxia-induced growth factor of vascular endothelial cells. It exists mainly in small and medium blood vessels. Its expression lasts for a long time and can protect the myocardium [15, 16]. In recent years, researchers discovered VEGF-B, VEGF-C, and VEGF-D. These VEGFs can bind to and activate VEGF receptors (VEGFR-1, VEGFR-2, and VEGFR-3), expressed in endothelial cells, and promote angiogenesis [17].

## 4. The Mechanism of Angiogenesis after Myocardial Infarction

**Figure 1:**
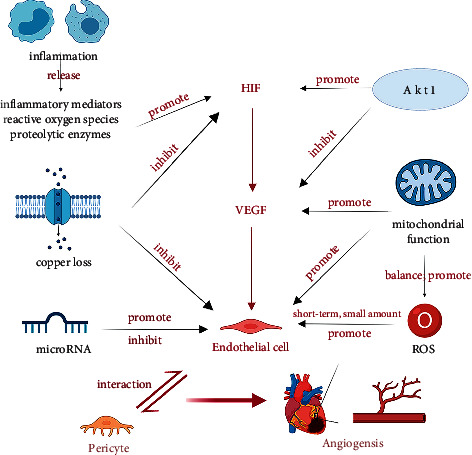
The mechanism of angiogenesis after MI. Copper loss, microRNA, AKT1, inflammation, reactive oxygen species (ROS), mitochondria, and the interaction between endothelial cells and pericytes play a role in angiogenesis.

Myocardial ischemia can induce cardiac angiogenesis, and its mechanisms are diverse, involving multiple factors and reactions. We systematically review the aspects of copper loss, microRNA, Akt1, inflammation, reactive oxygen species (ROS), mitochondria, and the interaction between endothelial cells and pericytes (Figure 1).

### 4.1. Copper Loss

The formation of a sufficient number of new blood vessels after myocardial ischemia largely depends on the upregulation of angiogenic genes such as VEGF. This requires that a certain amount of HIF-1a and HIF-2a accumulate in cardiomyocytes [18, 19]. Studies have confirmed that copper can affect the migration of endothelial cells, and supplementing copper in the daily diet can enhance the expression of VEGF and enhance angiogenesis [20]. However, myocardial ischemia leads to the loss of copper ions, reducing HIF-1 activity and thereby hindering angiogenesis and further aggravating myocardial damage [21].

Although copper can stabilize HIF-1*α* by inhibiting prolyl hydroxylase, this effect does not play a significant decisive role [22]. Therefore, the lack of copper does not affect the stability of HIF-1*α*. However, the role of copper has been highlighted in HIF-1 activation and transcription. First, the activation of HIF-1 requires the participation of copper and short interfering RNA targeting copper chaperone for superoxide dismutase 1 (CCS), both of which are indispensable. Second, insufficient copper content will reduce the binding of HIF-1 and p300, hinder the formation of the HIF-1 transcription complex, and fail to activate the target gene to perform its normal function. In addition, the binding of HIF-1 to hypoxia-responsive element (HRE) will also be inhibited [21].

The effect of copper on human endothelial cells is cell selective. In the presence of 500 *μ*M CuSO_4_, the number of human umbilical vein endothelial cells can double in 48 hours without serum. However, copper has almost no effect on other types of cells, such as intravascular smooth muscle cells. Similarly, other metal ions cannot cause endothelial cell proliferation and migration even at the same concentration as copper. It can be seen that copper can effectively induce the migration of human endothelial cells and that reducing the loss of copper under myocardial ischemia will be beneficial to angiogenesis [23].

### 4.2. MicroRNA

MicroRNAs (miRNAs) are small noncoding RNA that can regulate gene expression. Some miRNAs can affect endothelial cells, affect angiogenesis, and restore myocardial hemoperfusion, but their sources, targets, and pathways are all different [24].

Studies have confirmed that miR-329, miR-487b, miR-100, and other miRNAs can participate in the neovascularization after ischemia. Mice with hindlimb ischemia can restore hemoperfusion to the preinjury state within one week after anti-miR-329 treatment, which increases the number and density of capillaries and, at the same time, increases the diameter of blood vessels [25]. Similarly, inhibiting miR-100 can also improve the blood perfusion of muscles in the ischemic area. The miR17-92 cluster can also play a role in promoting angiogenesis. Its expression is upregulated in myocardial hypoxia, which reduces the release of antiangiogenic molecules [26, 27]. miR-214 is also highly expressed in the peripheral area of the infarct, inducing the secretion of VEGF-A and thereby promoting angiogenesis and reducing calcium overload [28–30].

In addition, in the process of using bone marrow mesenchymal stem cells (MSCs) to treat MI, the participation of in vitro miRNA is indispensable to jointly protect the ischemic myocardium. miR-126 is an important regulator that affects cell proliferation and differentiation processes related to angiogenesis [31, 32]. Upregulation of miR-126 can enhance the expression of VEGF, which is achieved by inhibiting Spred1 and PI3KR2 [33]. Reducing the expression of miR-377 or upregulating miR-210 and miR-424 can also contribute to the effect of VEGF, promoting endothelial cell migration and blood vessel formation [34, 35]. However, while miR-210 targets EPHA3 and miR-424 targets cullin-2 (CUL2), both of which are upregulated under hypoxic conditions, miR-210 acts on a variety of cell types but miR-424 only acts on endothelial cells, each having an angiogenic effect [36, 37]. In addition, miR-21, which is inseparable from the PTEN/Akt pathway, can also help angiogenesis when MSCs are used to treat MI [38]. These miRNAs can better promote angiogenesis and ensure blood and oxygen supply to the heart.

### 4.3. Akt1

Akt, also known as protein kinase B, is a serine-threonine kinase. Its main subtype, Akt1, is one of the important therapeutic targets for promoting angiogenesis after MI. Akt1 is a key signaling molecule of vascular endothelial cells that can control cell proliferation and differentiation and regulate the maturation and permeability of blood vessels [39, 40]. Whether Akt1 has a pro- or antiangiogenic effect depends on its amount and activation state.

It has been reported that Akt1 is not conducive to the activation of VEGF-A, and inhibiting its expression can contribute to the regeneration of the functional capillary network, increasing the number of capillaries that can support the myocardium and making it more powerful [40, 41]. The absence of Akt1 will enhance the expression of VEGF-A, promote angiogenesis, improve microcirculation, and, to some extent, compensate for the reduced blood flow of the coronary vascular system. However, the lack of Akt1 reduces cardiac function [42].

However, it is worth noting that the role of Akt after MI may be two-way. In the chronic phase of adaptive cardiac hypertrophy, Akt1 overexpression has been confirmed to reduce angiogenesis [43]. However, acute Akt1 overexpression can increase HIF-1*α* expression through the TSC1-TSC2/Rheb/mTORC1 pathway [44–46]. The Alt-PI3K pathway can also complete the migration of endothelial cells mediated by VEGF, and statins also promote the proliferation and differentiation of endothelial progenitor cells through this pathway, thereby promoting angiogenesis and protecting the heart under ischemia [45, 47, 48]. In contrast, Akt1 silencing significantly affects the adhesion of vascular smooth muscle cells to endothelial cells, impairs parietal cell coverage, and reduces angiogenesis buds [49]. On the other hand, Akt can phosphorylate endothelial nitric oxide synthase (eNOS), which is effective in angiogenesis induced by growth factors and angiotensin II [50, 51]. Although the overall effect of inhibiting Akt1 on the heart needs further study, its effect on angiogenesis is beyond doubt.

### 4.4. Inflammation and Inflammatory Cell Infiltration

Inflammation and inflammatory cell infiltration are very common in MI. Inflammatory cells such as neutrophils, lymphocytes, monocytes, and macrophages continuously infiltrate the myocardium damaged by ischemia, which can help angiogenesis. Among them, macrophages may be the main cell type that promotes angiogenesis after MI. To some extent, this process is achieved through the activation of the innate immune response by toll-like receptors (TLR) [52, 53].

Monocytes and macrophages can infiltrate the endangered ischemic myocardium, leading to the release of inflammatory mediators, ROS, and proteolytic enzymes and thereby promoting angiogenesis. In this process, the controlled recruitment of macrophages and the role of Ly-6Clow monocytes are involved [54–56]. Studies have shown that under the condition of oxidative stress, the hemoglobin released by the lysis of red blood cells can be combined with haptoglobin and internalized into macrophages. If such macrophages are relatively iron-deficient, that can lead to a HIF-*α*-mediated increase in VEGF levels increase and, consequently, more vigorous capillary angiogenesis [57, 58]. Other studies have also pointed out that macrophages, platelets, and fibroblasts can induce the expression of Thbs1 after MI. Thbs1 is an angiogenesis inhibitor that can resist the effects of VEGF and promote endothelial cell apoptosis, thereby exerting anti-angiogenesis properties and weakening the ability of fibroblasts to strongly stimulate angiogenesis. This result is particularly significant on the seventh day after MI. This indicates that some anti-inflammatory signals, such as TGF*β*1, can induce the expression of Thbs1 and hinder angiogenesis [59–61]. Inflammatory cells are also the main source of ROS. Another study pointed out that stem cell therapy for MI may be related to crosstalk between stem cells and macrophages [62]. In addition, the expression of some chemokines is also one of the factors that promote angiogenesis after MI, such as the upregulation of monocyte chemokine 1 (CCL2), which acts on the vascular endothelium [63, 64].

However, the different subgroups and functions of inflammatory cells may have two opposing effects on myocardial angiogenesis after infarction. Excessive inflammation will aggravate cardiac function damage and lead to ventricular remodeling [65, 66].

### 4.5. Oxidative Stress and ROS

Oxidative stress has both positive and negative effects on angiogenesis. ROS, which can be formed by endothelial cells and other vascular cells under hypoxia and can directly or indirectly connect with angiogenesis [67]. A small amount of short-term ROS can promote angiogenesis, but a large amount of long-term ROS can cause tissue damage [68, 69].

On the one hand, oxidative stress can mediate and regulate angiogenesis and play a promoting effect, which is especially true of hydrogen peroxide (H_2_O_2_) at a low concentration. VEGF completes the induction of endothelial cell proliferation and migration by increasing the content of ROS in the cell [70, 71]. In human umbilical vein endothelial cells (HUVECS), an appropriate increase of ROS can promote the formation of capillary tubes, but if NADPH oxidase inhibitors or free radical scavengers are added, the above effects will be inhibited. These experimental results are consistent and can support each other [72–74]. On the other hand, excessive oxidative stress will reduce the angiogenic activity of the ischemic myocardium, reduce the secretion of VEGF, and impair the proliferation, migration, and tube formation of endothelial progenitor cells, thus weakening and delaying angiogenesis. This is especially common in the case of MI complicated by diabetes. More severely, the endothelial cell damage caused by hydrogen peroxide exceeding 126 *μ*M is fatal [75, 76]. Similar to this view, the joint expression of hTK1 and hTIMP1 genes can weaken oxidative stress and contribute to the formation of new blood vessels [77].

### 4.6. Mitochondrial Function

After MI, the normal function of mitochondria is vital for improving myocardial ischemia. The proliferation and migration of endothelial cells, and even the renewal of blood vessels, depend to a certain extent on the adaptive response of mitochondria. Changes in the number, size, and morphology of mitochondria can change extracellular signals, thereby changing intracellular processes, which is the contribution of mitochondrial dynamics [78]. Since MI will cause the cells to be in a relatively hypoxic situation, the ability of mitochondria to move promotes the activation of VEGF, thus opening up angiogenesis after MI [79]. Mitochondria can also integrate environmental factors under hypoxia with the growth and migration of endothelial cells into a signal network [80]. In addition, as GTP hydrolyzes on the mitochondrial membrane, the two subtypes of mitotic fusion proteins, Mfn1 and Mfn2, have the ability to mediate mitochondrial outer membrane fusion [81] and can increase endothelial migration and angiogenesis under VEGF expression [82].

In terms of metabolism, although mitochondrial respiration in endothelial cells will be forced to decrease after MI, the process of glycolysis is greatly enhanced to compensate for the reduced energy production caused by decreased respiration. In addition to providing ATP for cells, the upregulation of glycolysis can also serve as a signal to increase the proliferation and migration of endothelial cells, which greatly promotes angiogenesis [83, 84].

On the other hand, mitochondria also play a major role in balancing the production and removal of ROS. As an oxidant, H_2_O_2_ is extremely stable and can cross cell membranes [85]. Therefore, when the H_2_O_2_ produced by mitochondria increases, its function as a signal molecule becomes prominent, which can activate a series of signals through the PAK, Akt, and ERK pathways so that endothelial cells can migrate and proliferate to ensure angiogenesis [71]. At the same time, reduced nicotinamide adenine dinucleotide phosphate oxidase complex (NOX-4) can be highly expressed in the mitochondria of endothelial cells, and the induced ROS signal also contributes to angiogenesis [78]. However, it is worth noting that the amount of ROS is not as great as is possible. If excessive, it will hinder the normal function of mitochondria. Therefore, some mitochondrial proteins called antioxidant defense proteins, such as manganese superoxide dismutase, thioredoxin 2, thioredoxin reductase 2, and uncoupling protein 2, are used to balance the amount of ROS [86–89].

It should not be overlooked that excessive angiogenesis will also be limited because mitochondria can control the degeneration of blood vessels by releasing proapoptotic molecules. Cytochrome c, Smac/DIABLO, Omi/HtrA2, and other substances can activate caspase, and the mechanism of cell degradation sets a “lifespan threshold” for endothelial cells, balancing the “coming and going” of the endothelium [90].

### 4.7. Interaction between Pericytes and Endothelial Cells

In the process of angiogenesis after MI, in addition to the participation of endothelial cells, the presence of pericytes is also essential. Pericytes are slender blood vessel wall supporting cells [91], distributed and extending along endothelial cells [92]. Pericytes can directly contact endothelial cells and interact with endothelial cells by sharing a basement membrane [93]. The interconnection and interaction between the two cell types provide conditions for their migration and proliferation. On this basis, they contribute to the regeneration and maturation of blood vessels and are essential for the development and function of normal blood vessels [94, 95]. Researchers have generally recognized that there are several subgroups of pericytes. Among them, the hematopoietic pericyte subgroup is most closely related to angiogenesis after MI and can promote angiogenesis, and the mesenchymal stem cell subgroup can promote blood vessel maturation [93].

In the early stage of angiogenesis, pericytes can degrade the extracellular matrix, rupture the basement membrane, and separate from endothelial cells. This is due to the binding of angiopoietin-1/2 from endothelial cells to Tie-2 receptors on pericytes. Thereby, signals are generated to induce pericytes to separate from the blood vessels to initiate angiogenesis [96–98]. After pericytes are detached, matrix metalloproteinases and proteases change the extracellular matrix, enabling pericytes to undergo cell migration [99]. VEGFR-A derived from pericytes can bind to VEGFR2 to ensure the proliferation and survival of endothelial cells and lead to the formation of new blood vessels [100]. To avoid continuous and unlimited endothelial cell proliferation, pericyte-derived transforming growth factor *β* can inhibit the increase in the number of endothelial cells [101]. After the formation of new blood vessels, pericytes return to the vicinity of endothelial cells to stabilize the new blood vessels under the action of platelet-derived growth factor-BB derived from the endothelium and heparin combined with an epidermal growth factor [102]. In addition, pericytes can also promote blood vessel maturation by covering new blood vessels [103]. Because pericytes can interact with N-cadherin, an adhesion molecule, the maturity and stability of newly germinated blood vessels are guaranteed [104]. After blood vessels are aged or damaged, pericytes will send signals through chemokine receptor 3 to weaken the process of blood vessel formation and promote blood vessel dissociation [105]. In summary, it can be shown that the crosstalk between endothelial cells and pericytes can regulate angiogenesis after MI, which provides a new basis for the treatment of myocardial infarction.

## 5. The Effects of Natural Herbal Medicines on Angiogenesis after Myocardial Infarction

Numerous studies have found that natural medicines are effective in treating MI. In recent years, a variety of herbs have also been shown to improve MI by promoting angiogenesis, and the mechanisms involved microRNA, Akt1, and the interaction between endothelial cells and pericytes.

### 5.1. *Salvia miltiorrhiza* Bunge Extracts

*Salvia miltiorrhiza* is an herbal medicine that has been used by humans for thousands of years. It has obvious effects on promoting the perfusion of ischemic myocardium and improving blood circulation (Zhu, 1998). Salvianolic acid B, tanshinone IIA, and sodium tanshinone IIA sulfonate are extracted from *Salvia miltiorrhiza* Bunge, whose medical parts are dry rhizomes and roofs [106].

Earlier, salvianolic acid B was found to promote angiogenesis by increasing the expression of VEGF, especially in the marginal zone of MI. Its effect is greater than that of benazepril, which is an important angiotensin-converting enzyme inhibitor in developed countries for preventing MI [107–109]. There have been reports in recent years that MSCs pretreated with salvianolic acid B have a better effect on promoting angiogenesis than those without Sal B treatment. This is because salvianolic acid B can promote the differentiation of MSCs into endothelial cells [110].

Tanshinone IIA can increase the expression of VEGF by increasing the expression of HIF-1*α* mRNA, resulting in increased angiogenesis in MI rats [111]. At the same time, the increase in VEGF expression can also promote the transformation of MSC to endothelial-like cells, increase the number of S-phase cells, and increase the tube-forming ability and proliferation ability of endothelial-like cells [112]. In addition, sodium tanshinone IIA sulfonate, a water-soluble derivative of tanshinone, may help promote angiogenesis and improve collateral circulation. Sodium tanshinone IIA sulfonate can increase *α*-SMA-positive and CD31-positive blood vessels, and the appearance of small new blood vessels is consistent with the result of increased VEGF expression [113].

### 5.2. *Carthamus tinctorius* L. Extracts

The herb *Carthamus tinctorius* L. has the effect of relieving myocardial ischemia and has long been used to treat MI [114]. Hydroxysafflower yellow A is the most critical active ingredient in *Carthamus tinctorius* L., which can promote ischemic myocardial angiogenesis. Hydroxysafflower yellow A can increase the number of endothelial progenitor cells in MI mice, promote cell migration and vascularization of HUVECS in a dose- and time-dependent manner, and increase the density of arterioles and capillaries. As far as the marker protein of angiogenesis is concerned, when hydroxysafflower yellow A doses are 30 and 60 mg/kg, the expression of VEGFR2 increases significantly, and when hydroxysafflower yellow A doses are 15, 30, and 60 mg/kg, the expression of *α*-smooth muscle actin is significantly upregulated. In addition, Hydroxysafflower yellow A can also enhance the expression of angiopoietin 1, Tie-2, VEGF-A, nucleolar protein, and matrix metalloproteinase 9 and increase the phosphorylation of Tie-2, Akt, and extracellular signal-regulated kinase 1/2. In recent years, some reports have reported that hydroxysafflower yellow A's proangiogenic effect depends on the Ang 1/Tie-2 signaling pathway, and other scientists have shown that hydroxysafflower yellow A can promote endothelial progenitor cell function through the HO-1/VEGF-A/SDF-1a signaling cascade. These all contribute to the formation of new blood vessels in the myocardium and further improve the heart function of MI mice [115–117].

### 5.3. *Pueraria lobata* Extracts

*Pueraria lobata* extract is extracted from a wild leguminous plant, *Pueraria lobata* (Willd.) Ohwi, which has been proven to promote angiogenesis in vivo and in vitro. *Pueraria lobata* is widely used in cardiovascular diseases such as angina pectoris and hypertension in China and can induce angiogenesis in the ischemic and nonischemic areas of MI models [118, 119]. When 80 mg/ml *Pueraria lobata* extract is used in the rat aortic ring or HUVECS, it induces a 5-fold increase in blood vessels at the edge of the aortic ring, which is consistent with the therapeutic effect of VEGF at a concentration of 20 ng/ml. However, it is worth noting that *Pueraria lobata* extract has not been found to increase the expression of VEGF, and its proangiogenic effect depends on MEK/ERK-, phosphatidylinositol 3-kinase/Akt/eNOS-, and Src/Fak-dependent pathways [120].

Puerarin is a flavonoid extracted from *Pueraria lobata* root that can increase the number of endothelial progenitor cells and promote angiogenesis. Its effects on improving coronary microcirculation and promoting angiogenesis have positive significance for the treatment of MI [121, 122]. In recent years, studies have shown that puerarin doses of 60 and 120 mg/kg can reduce the infarct size of the heart of rats with MI. When the dose of puerarin is 120 mg/kg, it can induce angiogenesis in the ischemic and nonischemic areas of the myocardium in the MI rat model and increase the number and area of myocardial capillaries. This indicates that puerarin has dual effects on increasing blood supply: one is to directly reduce the area of the infarcted myocardium at ischemic sites, and the other is to indirectly increase the blood supply to nonischemic areas to improve the function of compensatory hypertrophic myocardium. Its proangiogenic effect may be related to the induction of VEGF and/or eNOS expression [119]. Further studies have shown that puerarin exerts a compensatory angiogenic effect by upregulating the expression of key angiogenic factors VEGF-A, angiotensin 1, and angiotensin 2 in the setting of cardiac stress caused by MI [121].

### 5.4. Astragalus Extracts

Astragalus has been proven to increase blood supply [123]. The active ingredient astragaloside IV extracted from *Astragalus* can promote angiogenesis. The proliferation of HUVEC is related to the dose of astragaloside IV. Under the treatment of 100 *μ*g/ml astragaloside IV, the number of cells increased by 54%, the migration of vascular endothelial cells increased by 109%, and the gene expression level of VEGF increased nearly 2.5 times, effectively formed slender capillaries, and thus built a blood vessel network. However, astragaloside IV can only be effective when myocardial blood supply is insufficient and angiogenesis is impaired; its effects are not significant under physiological conditions [124, 125]. It is reported that this is because astragaloside IV can upregulate the expression of VEGF and promote angiogenesis through the PTEN/PI3K/Akt pathway, with an optimal concentration of 80 *μ*mol/L [126–128]. The angiogenic effect of astragaloside IV in rats with MI is achieved through the protein kinase D1-high-density lipoprotein receptor 5-VEGF pathway [129]. It is also reported that the angiogenic effect of astragaloside IV may be related to the upregulated expression of connexins Cx37, Cx40, and Cx43 and the enhancement of gap junction cell communication. When astragaloside IV and tanshinone IIA are used together, the effect is better [112]. In addition, the angiogenesis-promoting effect of *Astragalus* polysaccharide has also been confirmed by multiple studies. *Astragalus* polysaccharide can increase the blood flow of the skin near the wound in a dose-dependent manner and increase the density of microvessels [130]. Its angiogenesis-promoting function is at least in part through the VEGF/VEGFR and Ang-1/Tie-2 pathways [131].

### 5.5. *Panax ginseng* C.A. Mey. Extracts

Ginseng is the dried root of *Panax ginseng* C.A. Mey., a common herbal medicine in China, Japan, and Korea. Ginseng is believed to have vasodilation, anti-inflammatory, antioxidative stress, and other effects, which are effective for cardiovascular diseases, especially MI and subsequent angiogenesis [132]. The main bioactive component of ginseng is ginsenosides. Some of these triterpene saponins, such as ginsenosides Re and Rg1, have the effect of promoting angiogenesis. Ginsenosides f1 and Rh1 also have the ability to induce the migration and proliferation of endothelial cells [133].

Ginsenoside Re belongs to the ginsenosides, which can significantly increase the number of new capillaries and the content of tissue hemoglobin, which indicates that it has the effect of inducing angiogenesis and can resist MI [134]. As for in vitro experiments, ginsenoside Re can promote the proliferation, migration, and tube formation of HUVECS, just like basic fibroblast growth factor, but the effect of ginsenoside Re is relatively stable and hardly affected by temperature, pH, and solvent type [135]. Its effects are dose-dependent, with an optimum concentration of about 30 *μ*g/ml [134].

Ginsenoside Rg1, another major ginsenoside in *Panax ginseng* C.A. Mey., can mediate hypoxia-dependent HIF-1*α* upregulation and increase the expression of VEGF through the PI3K/Akt/mTOR pathway, thereby showing a significant angiogenic effect [136, 137]. Further studies have confirmed that Rg1 can attenuate the expression of miR-214 in HUVECs and increase the expression of eNOS, thereby increasing cell migration and tube formation in vitro [138, 139]. This is consistent with the view that ginsenoside Rg1 has estrogen-like activity and can act on endothelial cells to exert angiogenesis [140].

### 5.6. *Panax notoginseng* (Burkill) F.H. Chen Extracts

*Panax notoginseng* (Burkill) F.H. Chen is a popular herb; in addition to treating high blood pressure and dizziness, it also has angiogenesis effects and can improve acute MI [141]. Its main active ingredient is saponin. In vitro, *Panax notoginseng* extract can promote the proliferation of HUVECS and increase its exercise capacity while increasing the formation of capillary-like tube branches, which is achieved through AMPK and eNOS-dependent pathways [142]. In vivo, MI rats treated with PNF induced a nearly threefold increase in VEGF mRNA expression, accompanied by denser growth of blood vessels around MI. The formation of new blood vessels in these infarcted areas can relieve myocardial ischemia and save damaged cardiomyocytes [143]. This effect may be related to the upregulation of HIF-1, VEGF-A, and KDR gene expression [141]. What is more interesting is that the angiogenic effect of ginsenosides in PNF is dose-dependent. When a dose of FS 25 mg/kg/d is administered to rats, the density of new blood vessels can be better increased; if the dose is either exceeded or insufficient, the effect will be weakened, and even the opposite effect will be produced [143].

Other studies further show that some saponins isolated from *Panax notoginseng,* such as notoginsenosides Ft1 and R1, also have the effect of promoting angiogenesis and tube formation of HUVCEs. Notoginsenoside Ft1 promotes angiogenesis through HIF-1*α*-mediated VEGF secretion and regulation of PI3K/alkaline phosphatase and Raf/MEK/ERK signaling pathways [144], while notoginsenoside R1 can activate angiogenin 2/Iron 2 pathways achieve this effect [145].

### 5.7. *Cinnamomum cassia* (L.) J. Presl Extract

*Cinnamomum cassia* (L.) J. Presl bark can act on the vasculature of the human body, promote angiogenesis of the infarcted myocardium, and improve blood circulation in the coronary vasculature [146, 147]. Cinnamaldehyde is an essential oil separated from *Cinnamomum cassia* (L.) J. Presl, and it is one of the main effective ingredients to promote angiogenesis. Cinnamaldehyde can promote the proliferation, migration, and tube formation of HUVECS and increase the amount of VEGF secreted by HUVECS. Furthermore, cinnamaldehyde can repair a part of zebrafish internodal blood vessels pretreated with PTK787, a selective inhibitor of the VEGF receptor. In other in vivo experiments, cinnamaldehyde can promote capillary angiogenesis in mice and increase the thickness of blood vessel walls. Studies have shown that cinnamaldehyde can promote therapeutic angiogenesis after MI by activating PI3K/AKT and MAPK signaling pathways [148].

Cinnamic acid, another active compound of *Cinnamomum cassia* (L.) J. Presl, can promote the proliferation, migration, and differentiation of endothelial cells in vitro. In vivo, the effect and mechanism of cinnamic acid in promoting angiogenesis are similar to VEGF165, and its angiogenic activity depends on the expression of VEGF. Existing studies have confirmed that this is because cinnamic acid can promote angiogenesis by upregulating the expression of VEGF and Flk-1/KDR, thereby improving the lack of blood microcirculation [149].

### 5.8. *Rehmannia glutinosa* (Gaertn.) DC Extract

*Rehmannia glutinosa* (Gaertn.) DC, a plant of the Scrophulariaceae family, has the effect of promoting angiogenesis [1]. *Rehmannia glutinosa* (Gaertn.) DC extract can significantly reduce myocardial ischemia by promoting capillary angiogenesis in the second to fourth weeks after MI, the chronic phase. At the same time, the number of endothelial progenitor cells and their proliferation, migration, and tube formation capabilities are also improved. *Rehmannia glutinosa* (Gaertn.) DC extract is a safe endothelial progenitor cell agonist, with fewer adverse events such as increased vascular permeability and high restenosis rate [150]. *Rehmannia glutinosa* (Gaertn.) DC extract can also enhance the mobilization and migration of endothelial progenitor cells after MI by activating the SDF-1*α*/CXCR4 cascade, showing that RGE can promote capillary regeneration in the chronic phase of myocardial infarction [1, 151].

The components of *Rehmannia glutinosa* (Gaertn.) DC extract have not been fully studied, but studies have shown that catalpol is the main active ingredient. Catalpol can treat MI by improving the survival rate and VEGF secretion of transplanted bone marrow mesenchymal stem cells (BMSC) in ischemic myocardium. Catalpol-pretreated BMSC dose-dependently exerts a better significant angiogenic effect. This is because catalpol pretreatment contributes to the effect of BMSC transplantation to increase the expression of CD31 in ischemic myocardium [152].

### 5.9. Leonurus japonicus Houtt Extracts

*Leonurus japonicus* Houtt, also known as motherwort, belongs to the Labiatae family and has the effect of improving coronary blood flow and microcirculation [153, 154]. Leonurine, the main active component of *Leonurus*, can significantly induce the expression of survivin and VEGF in chronic myocardial ischemia, and the activation of HIF-1*α* mediated by it can promote angiogenesis in MI mice [155]. In vitro, leonurine can significantly enhance endothelial cell migration and tube formation during hypoxia. It can be seen that leonurine has the ability to reduce or eliminate myocardial ischemia by forming new blood vessels. More interestingly, leonurine can not only restore part of the blood perfusion and maintain tissue activity after tissue ischemia but also reduce mitochondrial dysfunction and promote VEGF upregulation, thereby protecting angiogenesis from age-dependent damage [156].

In addition, stachydrine, another representative alkaloid of leonurine, has also been confirmed to have significant biological activity that can enhance the angiogenesis of transgenic zebrafish embryos treated with sunitinib, an angiogenesis inhibitor [157]. Further studies have shown that stachydrine can promote the transformation of HUVECs to form new blood vessels. The molecular mechanism of promoting angiogenesis may be related to the activation of VEGFR2/MEK/ERK and the inhibition of the mitochondrial-mediated apoptosis signaling pathway, which is of great significance for therapeutic angiogenesis after MI [158].

### 5.10. *Scutellaria baicalensis* Georgi Extract

The root of *Scutellaria baicalensis* Georgi is the main part used in medicine; its water extract can promote angiogenesis at low doses (0.2 mg/ml) [159]. Baicalin, belonging to flavonoids, is one of the effective ingredients of *Scutellaria baicalensis*. When the concentration of baicalin is as low as 10 *μ*g/ml to 50 *μ*g/ml, it can strongly promote the expression of VEGF and effectively induce endothelial cell migration, leading to the promotion of angiogenesis. In addition, in the chick aortic arch assay, microvessels germinated after feeding with baicalin medium, further confirming that baicalin can induce angiogenesis. This result can be obtained by overactivating the ERR*α*/PGC-1a pathway. However, it is worth noting that high doses of baicalin can inhibit angiogenesis and aggravate ischemia. Therefore, it is necessary to pay special attention to the dual effects of different doses of baicalin on angiogenesis [159, 160].

### 5.11. *Geum macrophyllum* Willd. Extract

The extract of *Geum macrophyllum* Willd. has an important dual role in the early angiogenesis and myocardial formation of acute myocardial infarction. It can make many new blood vessels appear in the heart tissue 24–48 hours after infarction and limit infarct size by 35% to 45%. The emergence of these new functional blood vessels can promote the early reconstruction of the damaged blood supply network to slow down myocardial damage. In vitro, *Geum japonicum* can also increase the rapid revascularization of muscle injury in animal models within 24 hours, which further confirms the therapeutic angiogenic effect of *Geum japonicum* [161]. However, the mechanism underlying this result is unclear.

## 6. Clinical Study of Natural Herbs to Promote Angiogenesis after Myocardial Infarction

In clinical trials, Chinese herbal medicine is also effective in treating myocardial infarction. Bulbus allii macrostemi improves symptoms in patients with non-ST-segment elevation myocardial infarction by antioxidative and ox-LDL lowering. [162] Another study showed that, compared with conventional western medicine treatment, the combined application of western medicine and safflower injection can inhibit the expression of GP IIb/IIIa receptors, indicating that safflower can treat the acute coronary syndrome. [163] In addition, Danhong injection with safflower and salvia as the main active ingredients can also significantly improve the symptoms of angina pectoris in patients with coronary heart disease after pci, such as the frequency of angina pectoris, the degree of pain, and the dosage of nitroglycerin all decreased [164]. Other drugs composed of traditional Chinese medicines, such as Tongxinluo, Luofengning granule, Shexiang Baoxin Pill, Xinyue capsule, Jiuxin Pill, and Shuangshen Tongguan Capsule, have all shown improvement in myocardial infarction patients in various clinical trials. They can improve myocardial microcirculation perfusion and even improve no-reflow and infarct size. This is partly due to angiogenesis [165–170]. In the above clinical experiments, no obvious adverse events were found.

## 7. Conclusion

This review discusses the major effects of copper loss, noncoding RNA, Akt1, inflammation, ROS, mitochondria, and the interaction between endothelial cells and pericytes on angiogenesis after MI. We further explored the effects of 11 natural herbs on promoting angiogenesis. They can improve microcirculation and restore blood supply to ischemic areas, thereby alleviating MI. This conclusion has been repeatedly verified not only in animal experiments but also in clinical trials. Angina symptoms and no-reflow area were significantly improved in MI patients after herbal treatment (Table 1). In the future, natural proangiogenic drugs may become candidates for the treatment of cardiovascular diseases. Researchers should give them more attention, try to conduct large-scale animal experiments and clinical studies, deeply explore the synergy of multiple biologically active plant ingredients, and elaborate on the clinical effectiveness and safety of these natural herbs.

## Figures and Tables

**Table 1:** Detailed information about bioactive ingredients that promote angiogenesis.

Components	Source	Chemical formula	Biological activity	Target cells	References
Salvianolic acid B	*Salvia miltiorrhiza* Bunge	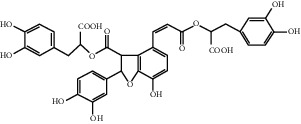	Enhance the expression of VEGF; promote the differentiation of mesenchymal stem cell into endothelium cells	Mesenchymal stem cells	[107–109]

Tanshinone IIA	*Salvia miltiorrhiza* Bunge	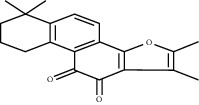	Promote the angiogenesis of mesenchymal stem cell-derived endothelial cell-like cells; enhance HIF-1*α* mRNA expression	Mesenchymal stem cells	[112, 113]

Sodium tanshinone IIA sulfonate	*Salvia miltiorrhiza* Bunge	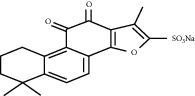	Promote the expression of VEGF	NA	[111]

Hydroxysafflor yellow A	*Carthamus tinctorius* L.	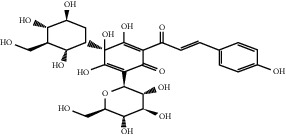	Enhance the expression of angiogenin 1, Tie-2, VEGF-A, nucleolin, and matrix metalloproteinase-9; increase the phosphorylation of Tie-2, Akt, and extracellular signal-regulated kinase 1/2	Endothelial cells	[116, 117]

*Pueraria lobata* extract	*Pueraria lobata*	NA	Activate MEK/ERK-, phosphatidylinositol 3-kinase/Akt/eNOS-, and Src/Fak-dependent pathways	Endothelial cells	[120]

Puerarin	*Pueraria lobata*	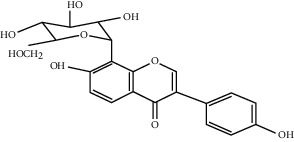	Upregulate the expression of key angiogenesis factors VEGF-A, angiotensin 1 and angiotensin 2	Endothelial cells	[121]

Astragaloside IV	*Astragalus*	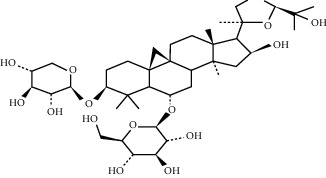	Through the PTEN/PI3K/Akt pathway; upregulate expression of Cx37, Cx40, and Cx43 and enhance gap junctional intercellular communication	Endothelial cells	[112, 126]

*Astragalus* polysaccharide	*Astragalus*	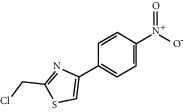	Through the VEGF/VEGFR and Ang-1/Tie-2 pathways	Endothelial cells	[130, 131]

Ginsenoside Re	*Panax ginseng* C.A. Mey.	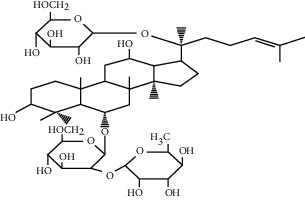	Promote the proliferation, migration, and tube formation of HUVECS	Endothelial cells	[134]

Ginsenoside Rg1	*Panax ginseng* C.A. Mey.	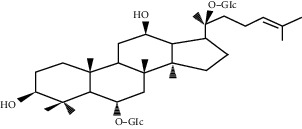	Mediate the hypoxia-independent upregulation of hypoxia-inducible factor-1a and increase the expression of VEGF	Endothelial cells	[136, 137]

Panax notoginseng (Burkill) F.H.Chen extract	Panax notoginseng (Burkill) F.H. Chen	NA	Upregulate the expression of HIF-1, VEGF-A, and KDR genes	Endothelial cells	[141–143]

Notoginsenoside Ft1	Panax notoginseng (Burkill) F.H. Chen	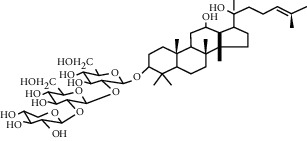	Promote angiogenesis via HIF-1*α* mediated VEGF secretion and the regulation of PI3K/AKT and Raf/MEK/ERK signaling pathways	Endothelial cells	[144]

Notoginsenoside R1	Panax notoginseng (Burkill) F.H. Chen	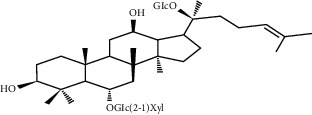	Activate the Ang2/Tie2 pathway to promote angiogenesis	Endothelial cells	[145]

Cinnamaldehyde	*Cinnamomum cassia* (L.) J. Presl	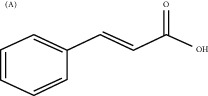	Activate PI3K/AKT and MAPK signaling pathways	Endothelial cells	[148]

Cinnamic acid	Cinnamomum cassia (L.) J. Presl	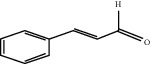	Upregulate the expression of VEGF and Flk-1/KDR	Endothelial cells	[149]

*Rehmannia glutinosa* (Gaertn.) DC. extract	*Rehmannia glutinosa* (Gaertn.) DC.	NA	Upregulate the expressions of angiogenesis-related ligands/receptors CD133, VEGFR2, SDF-1a, and CXCR4	Endothelial progenitor cells	[1]

Catalpol	*Rehmannia glutinosa* (Gaertn.) DC.	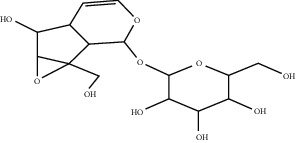	Upregulate the expression of VEGF	Bone marrow mesenchymal stem cells	[152]

Leonurine	Leonurus japonicus Houtt	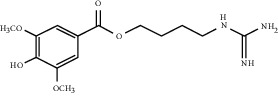	Induce the expression of survivin and VEGF during chronic myocardial ischemia	Endothelial cells	[155, 156]

Stachydrine	*Leonurus japonicu*s Houtt	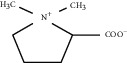	Activate VEGFR2/MEK/ERK to inhibit mitochondrial-mediated apoptosis signaling pathway	Endothelial cells	[157, 158]

Baicalin	*Scutellaria baicalensis*	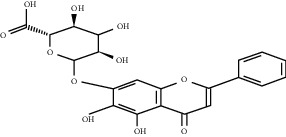	Upregulate the expression of several angiogenic genes and growth factors; overactivate the ERRa/PGC-1a pathway	Endothelial cells	[159, 160]

*Geum macrophyllum* Willd. extract	*Geum macrophyllum* Willd.	NA	Promote angiogenesis	NA	[161]

NA: not available; VEGF: vascular endothelial growth factor; HIF-1: hypoxia-inducible factor-1.

## Data Availability

No data were used in this article.
